# NX210c Demonstrates Therapeutic Potential to Restore Blood–Brain Barrier in a QSP Model of Relapsing–Remitting Multiple Sclerosis

**DOI:** 10.3390/ijms27031349

**Published:** 2026-01-29

**Authors:** Giulia Russo, Fianne Sips, Simona Catozzi, Pauline Bambury, Annette Janus, Mario Torchia, Valentina Di Salvatore, Luca Emili, Daniel Röshammar, Francesco Pappalardo, Yann Godfrin

**Affiliations:** 1 Department of Drug and Health Sciences, Università degli Studi di Catania, 95125 Catania, Italy; giulia.russo@unict.it (G.R.); valentinadisalvatore@unict.it (V.D.S.); francesco.pappalardo@unict.it (F.P.); 2 Mimesis Srl, 95125 Catania, Italy; 3 InSilicoTrials Technologies B.V., 5232 AC ‘s-Hertogenbosch, The Netherlands; fianne.sips@insilicotrials.com; 4 InSilicoTrials Technologies Spa, 34123 Trieste, Italy; simona.catozzi@insilicotrials.com (S.C.); pauline.bambury@insilicotrials.com (P.B.); mario.torchia@insilicotrials.com (M.T.); luca.emili@insilicotrials.com (L.E.); 5 Axoltis Pharma, 69008 Lyon, France; ajanus@axoltis.com (A.J.); ygodfrin@axoltis.com (Y.G.)

**Keywords:** blood–brain barrier, relapsing–remitting multiple sclerosis, NX210c, SCO-spondin, tight junction proteins, neuroinflammation, quantitative systems pharmacology, neurodegenerative diseases

## Abstract

Blood–brain barrier (BBB) breakdown is a hallmark of several neurological disorders, including multiple sclerosis (MS). NX210c, a novel therapeutic peptide, has shown promise in restoring BBB integrity, in both preclinical and clinical settings, offering potential for use in MS populations and across various central nervous system conditions with overlapping mechanisms. In this study, we evaluated the therapeutic potential of NX210c in patients with relapsing–remitting MS (RRMS) using a previous quantitative systems pharmacology (QSP) model currently redesigned to capture the dynamic interplay between BBB integrity and immune system activity. We validated the QSP model using both preclinical and clinical datasets, and generated virtual populations representing healthy individuals and RRMS patients for in silico testing. NX210c was assessed as both a monotherapy and in combination with established MS treatments. Simulations predicted time course changes in key BBB integrity markers, including tight junction protein (TJP) expression and transendothelial electrical resistance (TEER), under various dosing regimens. NX210c treatment was associated with a significant attenuation of BBB degradation compared to untreated controls (~7–8% higher TJP expression and BBB electrical resistance). Furthermore, we investigated the long-term impact of NX210c on clinical outcomes such as relapse rates. Both 5 and 10 mg/kg doses (single cycle [thrice-weekly for 4 weeks]) induced improvement in disease activity in RRMS patients, as well as a 10 mg/kg dose (single or repeated 4-week cycles every 6 months) in highly active patients. Particularly when administered alongside one of five commonly used MS therapies (interferon β-1a, teriflunomide, cladribine, natalizumab, ocrelizumab), in the highly active subpopulation, the model on average predicted a reduction in relapse frequency in the 10 mg NX210c-treated group versus untreated group from four to no relapses over two years. These findings suggest that NX210c may enhance therapeutic efficacy in RRMS by promoting BBB restoration and modulating immune responses, offering a promising avenue for combination treatment strategies.

## 1. Introduction

Neurodegenerative disorders, including Alzheimer’s disease, Parkinson’s disease, Amyotrophic Lateral Sclerosis, and multiple sclerosis (MS), impact large segments of the population. While there is great diversity in disease progression and functional impairment, due to variations in the affected brain regions, they share common pathological mechanisms at the cellular and molecular level [1,2,3,4]. Hallmarks of neurodegenerative diseases include protein misfolding and aggregation, mitochondrial dysfunction and oxidative stress, neuroinflammation, and blood–brain barrier (BBB) dysfunction [1,5,6,7,8].

The BBB serves as a highly selective barrier between the bloodstream and the brain, tightly regulating the central nervous system (CNS) environment and safeguarding critical neurological structures from potentially harmful endogenous and exogenous substances [5]. It is primarily composed of brain microvascular endothelial cells (BMECs), which are closely connected by tight junction protein (TJP) complexes. These include key proteins such as claudin-5, occludin, and ZO-1, as well as supporting components like pericytes, astrocyte endfeet, and the basement membrane. Together, they ensure the strict control of CNS with low paracellular permeability and entry only via transporter proteins. BBB integrity, however, declines progressively with aging, and becomes compromised in many neurodegenerative diseases [5,9]. Therefore, therapeutic strategies aimed at restoring BBB function hold significant promise for the treatment and management of CNS disorders [10,11,12].

NX210c is an innovative peptide derived from subcommissural organ (SCO)-spondin (an endogenous glycoprotein brain-secreted during embryogenesis). This drug candidate has demonstrated driving BBB restoration through TJ claudin-5, as well as neuroprotective effects and the ability to enhance glutamatergic synaptic transmission [13]. It has shown promising biomarker modulation and functional benefits in several preclinical and early clinical studies [13,14]. In particular, enhanced excitatory neurotransmission was observed as a result of the NX210c-induced increase in AMPA and GluN2A-containing NMDA receptor-mediated synaptic currents. These effects translated into restored memory in animal models with synaptic dysfunction, suggesting potential for treating CNS disorders associated with impaired glutamatergic signaling [15]. The mechanism of action (MoA) of NX210c was studied in mouse brain slice electrophysiology and behavioral models, confirming memory restoration after NMDAR antagonist treatment [13]. Functional recovery and tissue repair were demonstrated in a rat spinal cord injury model, showing improved motor function, bladder control, white matter preservation, and neuronal counts [16]. In healthy volunteers, NX210c exposure proved to be safe and well tolerated, with single doses ranging from 0.4 to 10 mg/kg, with only mild, transient adverse events [14]. Most recently, NX210c dosing cycles of thrice-weekly administration at 5 and 10 mg/kg in healthy elderly volunteers were shown to have a favorable safety profile and induce dose-dependent responses on blood biomarkers related to BBB integrity, including claudin-5, NfL, and SPARCL1 [17].

A key question in the development of compounds targeting neurodegenerative diseases is the selection of primary indications to prioritize. While in vivo and in vitro methodologies can offer insights, they do not provide a direct readout of the compound’s potential treatment effect across various patient populations, as they fail to capture the complexity and variability present in the clinical setting. Prioritizing indications where the greatest patient benefit can be achieved allows accelerating timelines and saving resources, while also optimizing patient exposure—minimizing unnecessary use of placebo or suboptimal treatments.

Quantitative systems pharmacology (QSP) approaches are increasingly being recognized as regulatory-acceptable tools to support drug development and submissions based on in silico simulations, especially in early-development stages to inform internal decision-making [18,19,20,21,22,23,24]. Such strategies leverage mechanism-based mathematical models that integrate drug pharmacokinetics (PK), pharmacodynamics (PD), and disease pathology to predict drug effects on biomarkers and clinical outcomes. They are suited to answering a wide variety of questions underlying key clinical decisions, such as investigating the potential of combination therapies, identifying and analyzing biomarker responses, expanding indications, and assessing how genetic and phenotypic variability within a population may influence expected treatment effects [25,26].

To evaluate the potential of NX210c in relapsing–remitting MS, simulate the age-related decline in the BBB, and predict NX210c’s therapeutic effect on restoring BBB integrity, we built upon the previously published MS TreatSim framework for RRMS disease dynamics [27,28,29]. Here, we have introduced a novel mechanistic module explicitly representing BBB integrity through tight junction protein dynamics and permeability proxies. Importantly, BBB integrity was mechanistically coupled to immune cell trafficking into the central nervous system, enabling the integrated evaluation of BBB-targeted therapeutic interventions and downstream relapse-related outcomes. This module was informed by preclinical and clinical data on NX210c’s MoA. Although this is not fully elucidated, previous studies have shown that the peptide enhances TJP expression and improves endothelial barrier stability, leading to reduced permeability. These downstream effects are consistent with the observed mitigation of relapse-related oligodendrocyte damage in the present model, due to decreased likelihood of autoimmune cell infiltration into the CNS. MS TreatSim builds upon a generalized immune system simulator (UISS), incorporates an RRMS-specific disease layer, and includes a selection of standard-of-care (SoC) treatment options for RRMS via their incorporated MoA and PK. Previous validation studies of the SoC treatments have confirmed MS TreatSim’s predictive performance with respect to both qualitative trends and quantitative treatment outcomes [28,29]. The present QSP model was finally leveraged to perform in silico clinical trial simulations assessing the potential of NX210c in RRMS patients as a monotherapy or in combination with different SoC, in terms of reducing relapse rates.

## 2. Results

### 2.1. Model Development and Validation

#### 2.1.1. The Blood–Brain Barrier Model and NX210c Pharmacodynamic Effects

We developed a mechanistic QSP model of the BBB that accounts for key features of age- and disease-related dysfunction, namely decreased expression of TJPs, reduced BMEC density, and impaired vessel permeability via BMEC–pericyte interactions. Notably, the model describes changes in TJP expression, as well as transendothelial electrical resistance (TEER) as a metric of permeability.

**Figure 1:**
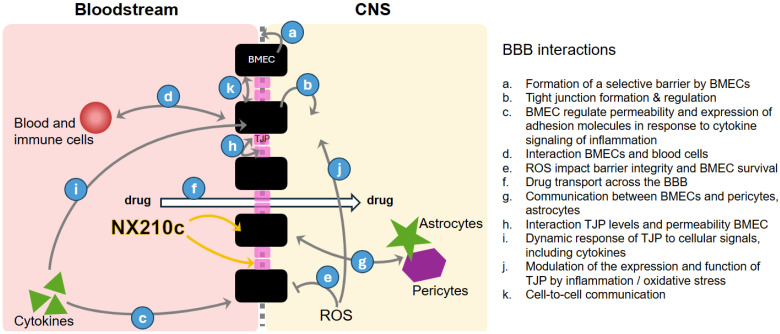
Schematic of the blood–brain barrier (BBB) model showing brain microvascular endothelial cells (BMECs) forming tight junctions (TJPs) and expressing transporters that regulate molecular exchange between the bloodstream and the central nervous system. Pericytes are embedded in the basement membrane and astrocytic endfeet surround the vessels, providing structural and signaling support. Cytokines and immune cell trafficking illustrate communication across the BBB. Color coding distinguishes bloodstream (light red), BBB interface (pink/grey), and brain parenchyma (yellow) compartments. Arrows illustrate the BBB cell interactions described on the right.

The pharmacodynamic effects of NX210c on BBB restoration were implemented through its impact on the TJP levels of claudin-5, BMECs, and TEER. Schematic representation of the BBB model depicting the key compartments, cell types, and molecular processes is displayed in Figure 1.

#### 2.1.2. Validation of NX210c Mechanism of Action

Preclinical in vitro and in vivo experiments demonstrated that NX210c increases the expression of TJPs (e.g., claudin-5 and occludin) in endothelial cells and reduces barrier permeability.

Our BBB model integrated with NX210c’s MoA was validated using in vitro data measured on a BMEC monolayer in mice [30]. In those experiments, the exposure to NX210c (1, 10, 100 μM) or vehicle for 4 h to 5 days was assessed on tight junction protein levels, dextran permeability, and TEER.

**Figure 2:**
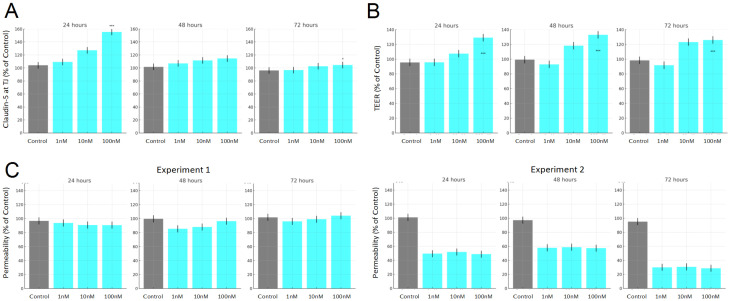
Predicted effects of NX210c on TJPs, TEER, and permeability, at varying doses (1, 10, and 100 mM). (**A**) Surface expression of claudin-5 in mouse brain endothelial cells, with and without NX210c. (**B**) TEER measurements across mouse BMEC monolayers. (**C**) Permeability of the brain endothelial monolayer following NX210c treatment.

Our simulations were able to capture the effect of NX210c on claudin-5 expression and TEER, reflecting enhanced barrier integrity and reduced BMEC permeability (Figure 2).

#### 2.1.3. Clinical Pharmacokinetic Predictions After NX210 and NX210c Injection

The linear form of the peptide (NX210) that was initially developed proved to rapidly undergo cyclization upon exposure to blood. Therefore, this spontaneous conversion implies that only the PK profile of the cyclic form (NX210c) can be effectively detected.

**Figure 3:**
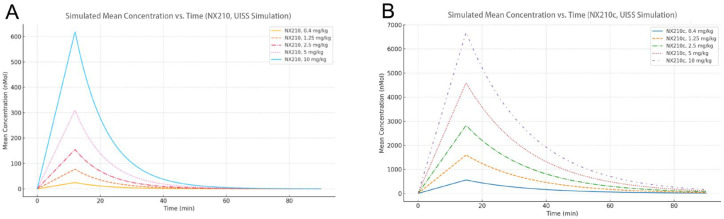
Simulated mean concentration–time profiles following single-dose administration of (**A**) NX210 or (**B**) NX210c, at escalating doses of 0.4, 1.25, 2.5, 5, and 10 mg/kg. The drug is administered via intravenous infusion, which was initiated at t = 0.

Our simulated drug concentration–time curves reproduced the observed PK in healthy individuals treated with NX210 in the SAD study [14] (Figure 3A) and the healthy elderly administered NX210c in the MAD study [17] interestingly predicted a 10-fold higher drug exposure with NX210c administration (Figure 3B).

#### 2.1.4. Integration with the MS TreatSim Platform

**Figure 4:**
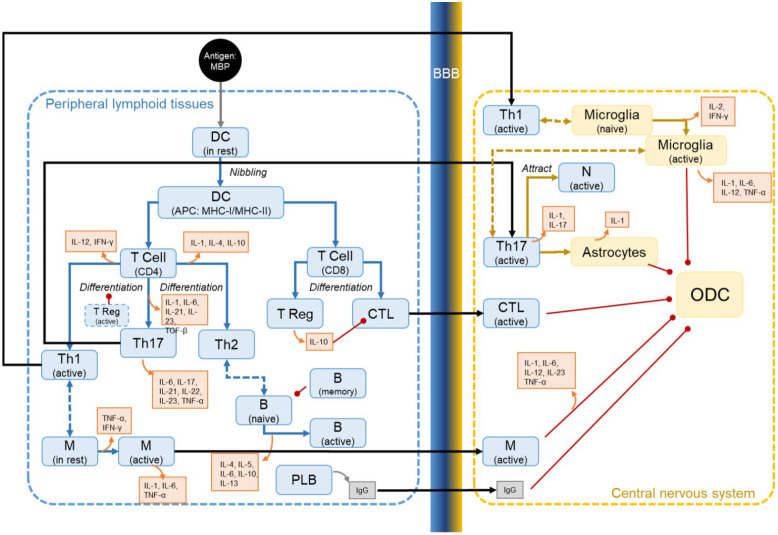
Final QSP model integrating the BBB model and NX210c’s MoA with MS TreatSim v2.0 for simulations of in silico clinical trials in RRMS virtual patients. The BBB-RRMS QSP model was built on immune system physiology as depicted. It captures the dynamics of key immune cell populations and their interactions in peripheral compartments (lymphoid tissues and CNS). In particular, antigen-presenting cells such as dendritic cells (DCs) activate naïve T cells, which differentiate into various effector and regulatory subsets (Th1, Th2, Th17, Treg, CTLs) and have an effect on microglia and oligodendrocytes (ODC). B cell activation and antibody production are also included. The BBB mechanistic model sketched here is detailed in Figure 1. Arrows show the cell-to-cell interactions.

The BBB model, together with the PK and PD of NX210c, was integrated with the MS TreatSim simulation platform, considering immune components contributing to CNS inflammation via infiltration across the BBB (Figure 4), and was leveraged to evaluate NX210c’s therapeutic potential in RRMS patients.

### 2.2. Model Simulations

#### 2.2.1. NX210c Treatment Is Predicted to Slow BBB Decline or Disruption in Healthy Subjects and RRMS Patients

**Figure 5:**
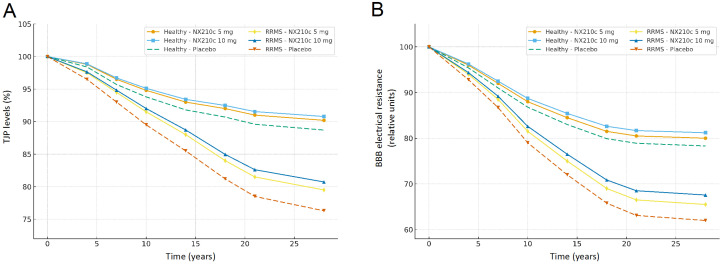
Simulation of NX210c pharmacodynamic effects on (**A**) TJP levels and (**B**) BBB electrical resistance, in 40-year-old healthy or RRMS subjects over a 30-year timeframe, with and without treatment, for different dose levels (5 or 10 mg/kg) after one single cycle.

The predicted long-term effects of NX210c on TJP expression and BBB electrical resistance (surrogate index of BBB integrity) have been simulated in both healthy and RRMS virtual populations (see Methods for details, Figure 5). NX210c demonstrated superior efficacy compared to NX210 at equivalent doses, suggesting the enhanced therapeutic potential of the cyclic peptide formulation, which is consistent with the higher PK concentrations observed for the active compound (Figure 3). The results show a faster decline in BBB integrity in untreated RRMS patients as compared to untreated healthy subjects: ~15% lower TJP or electrical resistance levels, within 30 years. Moreover, a dose-dependent protective effect of NX210c, i.e., mitigating the decline in BBB function over time, is observed in both healthy and RRMS populations, compared to the respective placebo group. Namely, up to ~7% (~2%) higher TJP expression and ~8% (~4%) higher BBB electrical resistance are predicted in RRMS (healthy) subjects treated with 10 mg/kg NX210c.

#### 2.2.2. NX210c Single-Dose Administration Reduces Disease Activity in Virtual RRMS Patients

**Figure 6:**
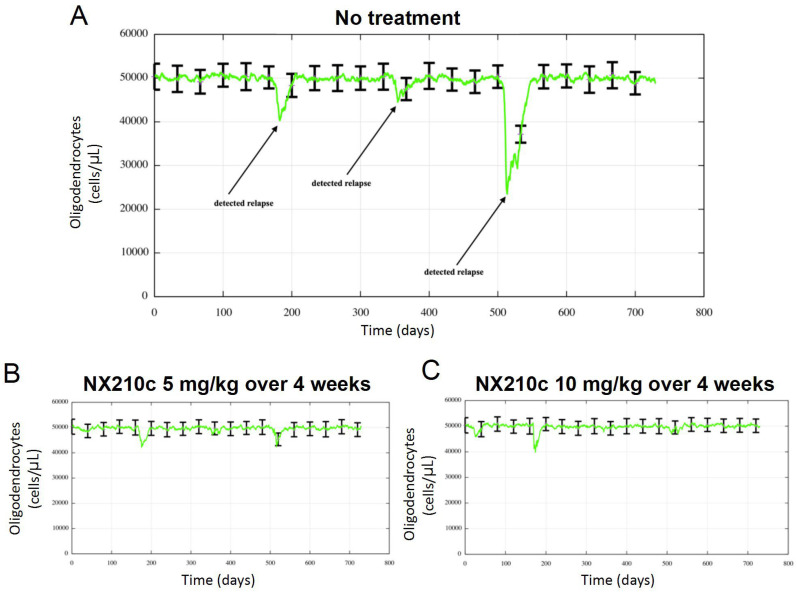
(**A**) Mean (green line) with variability (black bars) relapse dynamics over 2 years in a virtual population of 100 untreated RRMS patients. Relapses are quantified by a transient decrease in the oligodendrocyte population size, as pointed out by the arrows. (**B**,**C**) Predicted reduction in relapse frequency and amplitude following a single 4-week treatment cycle of NX210c at (**B**) 5 and (**C**) 10 mg/kg, thrice-weekly, in the virtual population.

The simulation platform was further leveraged to evaluate NX210c therapeutic potential in RRMS in terms of disease activity, characterized by relapse dynamics. Biological relapse was linked to sharp drops in the oligodendrocyte (ODC) population (Figure 6A) due to an increase in inflammatory cell trafficking to the CNS. Relapse severity was categorized as clinical or subclinical, whether ODC decreased, respectively, below 42,500 cells/μL, or above (see Section 4 ).

In the untreated group, the population exhibited a mean of approximately three cumulative relapses (clinical and subclinical) over a 2-year simulation period (Figure 6A). A single treatment cycle of NX210c (as studied in a previous Phase 1b clinical trial [17]) led to a notable (near-complete) reduction in relapse frequency across both evaluated dose levels (5 and 10 mg/kg), indicating clinically meaningful efficacy (Figure 6B,C).

#### 2.2.3. Repeated in Silico Treatment Cycles of NX210c Alleviate RRMS Long-Term Symptoms

**Figure 7:**
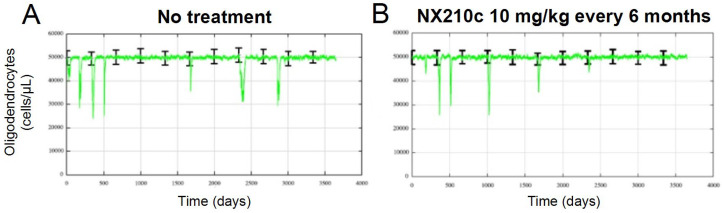
Mean (green line) with variability (black bars) simulated relapse activity in a virtual population of highly active RRMS patients, assessed via oligodendrocyte dynamics over 10 years. (**A**) Relapse activity without treatment. (**B**) Relapse activity following repeated NX210c treatment cycles (consisting of thrice-weekly administration of 10 mg/kg for 4 weeks), every 6 months.

Focusing on a patient population with highly active RRMS, we compared the results between untreated and treated groups (Figure 7). Notably, in the absence of treatment, patients exhibited frequent and severe clinical relapses, consistent with a highly active disease profile (Figure 7A). In contrast, repeated NX210c dosing cycles (every 6 months) led to a substantial and sustained reduction in both the frequency and severity of clinical relapses over the simulated decade: from seven to four clinical relapses (+2 subclinical). Additionally, the long-term impact on disease activity is shown, since 5 years after starting the treatment, the mean patient population result is almost clinical relapse-free (Figure 7B).

These findings overall suggest that cyclical NX210c administration may have durable disease-modifying effects in patients with high disease activity, supporting its candidacy as a maintenance therapy in RRMS. It is worth noting that the comparable intensity of breakthrough relapses observed under and without treatment reflects the fact that, in the current model formulation, NX210c primarily modulates relapse occurrence rather than the downstream inflammatory cascade once a relapse is triggered. Consequently, while relapse frequency and time in remission are substantially improved, the severity of the breakthrough events is not expected to change markedly. Clinically, this corresponds to a reduction in overall disease burden driven by fewer relapses, even if the intensity of isolated events remains similar.

#### 2.2.4. Synergistic Potential of NX210c in Combination with Standard of Care

**Figure 8:**
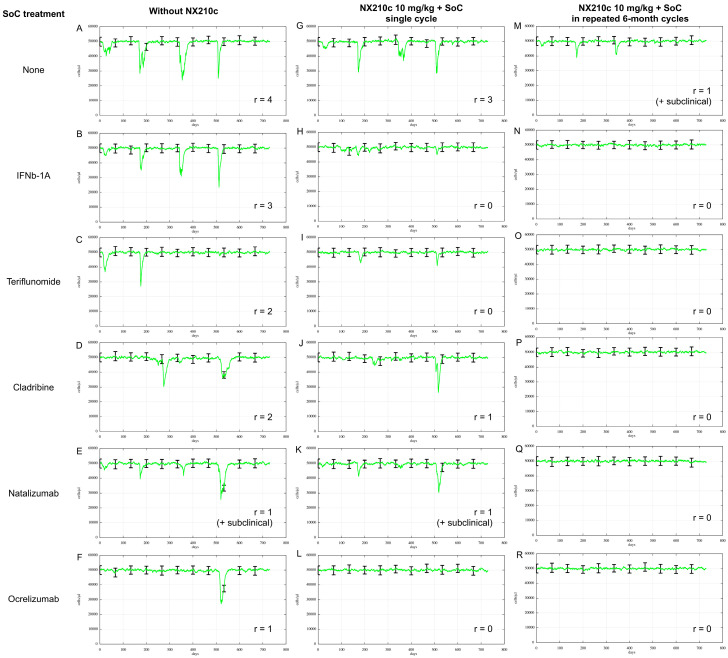
Mean (green line) with variability (black bars) predicted relapse rates over 2 years, for five standard-of-care treatments (interferon β-1a, teriflunomide, cladribine, natalizumab, ocrelizumab) and NX210c, in highly active RRMS virtual patients. (**A**) Untreated group. (**G**,**M**) NX210c monotherapy treatment group after (**G**) one single cycle, or (**M**) repeated 6-month cycles. (**B**–**F**) SoC monotherapy treatment groups. (**H**–**L**) Combination therapy treatment groups with one cycle of NX210c. (**N**–**R**) Combination therapy treatment groups with 6-month repeated NX210c dosing. Typical dosing regimens for each SoC therapy are detailed in Methods. One cycle of NX210 consists of 10 mg/kg thrice-weekly for 4 weeks. The number of relapses (r) is indicated for each scenario.

To explore the therapeutic potential of NX210c in combination with SoC, simulations were performed in the highly active RRMS population. Each simulation evaluated relapse activity under SoC monotherapy or combined with an NX210c regimen (Figure 8).

The results reproduced the known efficacy hierarchy among SoC treatments, confirming the model’s predictive performance. The virtual population treated with a single NX210c treatment cycle showed only minor benefit with respect to the untreated group (three versus four clinical relapses within two years), whereas repeated 6-month cycles of NX210c dosing proved more effective in reducing the relapse rate (one clinical relapse within two years). Moreover, when NX210c was administered in combination with the current SoC, the simulations predicted a consistent reduction in the relapse rate (one or none). In many cases, this resulted in extended periods of remission and near-complete suppression of relapse activity, suggesting a synergistic effect between NX210c and SoC agents.

## 3. Discussion

Here, through the use of a mechanistic QSP model, we demonstrated the potential of NX210c to reduce BBB breakdown. Our approach incorporated multiple data sources (internal and external, including in vivo, in vitro, and clinical datasets) within a mechanistic mathematical modeling platform to generate hypothesis-driven insights on the potential therapeutic use of NX210c. Following the integration of the novel BBB component within the RRMS QSP model, we showed that NX210c may have potential in RRMS, either as a monotherapy or as a synergistic combination therapy. Nevertheless, these findings will help derisking and optimizing the further clinical steps of the drug development.

In particular, simulations indicated that NX210c might improve BBB integrity. Key metrics such as TJP levels and BBB electrical resistance were consistently increased in virtual patients treated with NX210c, demonstrating a marked stabilization of tight junctions translating into reduced permeability. The impact of NX210c in reducing BBB leakage under both inflammatory and oxidative stress conditions was profound, suggesting a strong protective effect against CNS infiltration by peripheral immune cells.

These results illustrate the potential therapeutic application of NX210c in the treatment of (highly active) RRMS patients. From previous studies, NX210c appears to exhibit a multi-modal mechanism of action: by engaging beta-1 integrin-mediated signaling, it promotes neuronal survival and synaptic function; simultaneously, it enhances BBB and neurovascular barrier integrity by upregulating tight junction proteins and reducing permeability. A major advantage of NX210c in this context is precisely the targeting of BBB integrity rather than immune suppression—this completely distinct secondary MoA may allow increased efficacy without parallel increased risk of immune-derived adverse events. This drug reduces immune cell infiltration into the CNS (which is a key aspect of the pathology) and protects oligodendrocytes from inflammatory damage. Consequently, improved BBB maintenance through NX210c treatment could translate into a lower relapse rate by limiting ODC loss during inflammatory episodes.

The BBB model, combined with MS TreatSim, provided a powerful tool for simulating the effects of NX210c in both healthy subjects and RRMS patients, by integrating detailed physiological, cellular, and molecular components. It has been primarily used for mechanistic plausibility assessment and comparative scenario analysis. Its robustness was demonstrated by the consistency of treatment effects across heterogeneous virtual patients and across multiple endpoints (BBB integrity markers and relapse-related outputs). It offered insights into possible multi-agent therapies, where NX210c could be used alongside existing RRMS treatments to enhance BBB protection and reduce neuroinflammation. Furthermore, our approach highlighted NX210c’s promise in mitigating disease progression in other neuroinflammatory and neurodegenerative conditions, paving the way for future preclinical and clinical investigations.

Importantly, in our model, NX210c’s mechanism was implemented so that relapses that were predominantly driven by BBB permeability alterations were preferentially prevented. The remaining relapses observed in the treated groups therefore correspond to a subset of virtual patients characterized by stronger intrinsic autoimmune activation, for whom relapse triggering is less BBB-limited. This outcome reflects differential patient-level susceptibility and indicates an emergent responder/non-responder structure within the virtual cohort that motivates stratification strategies based on baseline inflammatory or BBB-related features.

When applying QSP models for the purposes of indication selection, the underlying data, model, and assumptions must be critically assessed for quality and suitability [31,32]. A key limitation of the current work is the assumption that clinical relapses are triggered by sudden inflammatory events only, thus ignoring part of the complexity of the MS pathology. Moreover, the observation period available for NX210c administration is relatively short, which constrains the ability to extrapolate predictions to long-term treatment regimens. These extrapolations should therefore be interpreted cautiously until supported by additional clinical evidence. Ongoing and future trials in neurodegenerative diseases are expected to provide extended longitudinal data to further refine and validate the model.

While the present modeling framework offers valuable mechanistic population-level insights on the translation of early biomarkers of effect into downstream clinical response, it is important to recognize the inherent limitations of QSP approaches, particularly when applied early in development. The current model was not designed to provide exact precise quantification of the magnitude of NX210c’s treatment effects, nor to fully characterize the range of physiological variability or uncertainty across the RRMS population. Instead, the simulations should be interpreted as qualitative hypothesis-generating outputs that highlight biologically plausible trends and support the rationale for further clinical evaluation guiding early-development go/no-go decisions, rather than as an exact quantitative approach for dosing optimization. As additional preclinical and clinical data become available, future work should incorporate more quantitative, data-rich modeling strategies to increase predictive precision and strengthen the model’s ability to inform later-stage development.

Therefore, the combined body of evidence and data integrated into the model provides a foundation for predictions and key opportunities for future investigation and clinical experimental design for NX210c, including the following:

*Optimization of Dosing Regimens:* The simulations allow for the evaluation of different dosing regimens for NX210c. Sensitivity analyses indicated that lower, more frequent doses could provide sustained BBB protection with reduced side effects. This approach may help refine clinical trial designs by focusing on optimal dosing that maximizes therapeutic benefits while minimizing risks.

*Patient Selection:* Using the in silico platform, it is possible to identify specific patient subgroups that might derive the most benefit from NX210c treatment. Notably, patients with high baseline BBB permeability or those with frequent relapse histories were shown to benefit greatly from NX210c, suggesting the importance of patient stratification in future clinical trials to optimize outcomes.

*Regulatory and Ethical Considerations:* The use of UISS-MS as an in silico trial platform aligns with current regulatory trends encouraging the adoption of computational models to support drug development. By reducing the reliance on animal models and optimizing early-stage clinical studies, these in silico trials contribute to ethical research practices, adhering to the 3Rs (Replacement, Reduction, Refinement) in biomedical research.

*Mechanistic Insights for Combination Therapy:* The data from combination therapy simulations suggest that NX210c could significantly enhance the effectiveness of existing RRMS treatments. Such insights are invaluable for designing future clinical trials, where combination therapy could be a strategy for patients who do not fully respond to monotherapy.

Over the last decade, the applications of QSP have grown quickly. While often used to gain insight and support internal decision-making, QSP simulations are also increasingly included in regulatory submissions [33], and the number of indications for which QSP models are available is rapidly expanding [34]. Here, we apply the established RRMS QSP to evaluate a novel indication for NX210c, ultimately reducing the development time, costs, and patient burden. Such a strategy can be a valuable tool in the development of therapeutics for neurodegenerative diseases, as they share many common features and therapeutics may have potential in multiple conditions.

## 4. Materials and Methods

### 4.1. Model Development

We developed a QSP model of the BBB function, using an agent-based approach implemented in the Universal Immune System Simulator (UISS) framework. The UISS is a multifunctional immune system simulator that was validated and verified across various immune-related indications, such as MS. Its MS-specialized simulator (MS TreatSim) was previously calibrated with the literature data describing the in vitro, in vivo, and clinical behavior of RRMS across different therapeutic interventions, and extensively validated both at individual and population levels [27,28,29,35].

The novel BBB integrity module extension, which incorporated NX210c’s drug action, enabled mechanistic and translational questions that could not be addressed previously. This integration was validated against preclinical data from prior studies in rats and mice [13,36] and supported by in vitro experimental evidence on tight junction protein modulation and permeability changes [30]. To allow the prediction of drug effects on clinically relevant outcomes, the integrative BBB–RRMS model design included key (i) physiology, (ii) pathophysiology, and (iii) treatment characteristics, inherited from the previously validated MS TreatSim framework. In particular, (i) the physiology layer encompassed anatomical compartments, cell types, molecules, protein expression levels, age dependency, immune system repertoire, and immune memory. (ii) The disease layer included the key processes involved in disease initiation and progression, which may affect the BBB. (iii) The treatment layer described the drug concentrations and mechanisms of action (MoA) of the drug at the cellular level.

To assess the potential effects of NX210c in RRMS patients, the BBB model was combined with the multiple sclerosis simulator MS TreatSim (described below). Inter-patient variability was inherently captured through the use of virtual populations within the model.

### 4.2. BBB Physiology

The major structural components of the BBB and its barrier function were represented by BMECs and TJP levels. We then connected these with several other agents of the BBB, according to the literature [37,38,39,40,41,42,43,44,45,46,47,48]. The implemented interactions include astrocyte-secreted factors (e.g., glial-derived neurotrophic factor), pericyte–endothelial signaling on BBB stability, changes in tight junction integrity, upregulation of adhesion molecules, endothelial activation, and oxidative stress response.

The BBB barrier function was described by incorporating mechanisms for paracellular and transcellular transport, controlling the passage of substances between the bloodstream and the brain and accounting for the influence of TJP dynamics and endothelial vesicular trafficking. The transendothelial electrical resistance (TEER), a functional readout of barrier tightness, was also modeled. A key determinant of BBB permeability in the model is the regulation of TJPs. The BBB model regulates the transportation of both agents and drugs across the barrier.

### 4.3. BBB Pathophysiology

The effect of aging on BBB integrity was implemented in the model following [49].

Normal aging is associated with increased BBB leakage as characterized by the ratio between cerebrospinal fluid and serum albumin concentrations (Qalb), as confirmed in a recent meta-analysis [50]. Sensitive neuroimaging studies have documented an age-related reduction in global and regional cerebral blood flow at the rate of 4 mL/min per year, along with decreased cerebral metabolic rates for oxygen and glucose and a lower cerebral blood volume.

### 4.4. NX210c Effects on BBB Function

The mechanism of action of NX210c for restoring BBB function was integrated into the model based on a variety of sources, including in vitro, in vivo, and clinical data, as detailed in the following subsections.

#### 4.4.1. NX210c Pharmacokinetics

The implementation of the PK model for the drug was informed by clinical single and multiple ascending dose studies [14,17]. In each cohort of the single ascending dose study (EudraCT registration identifier: 2020-000859-12), 6 healthy subjects were administered an intravenous dose of either 0.4, 1.25, 2.5, 5, or 10 mg NX210. The compound then transformed into its cyclic version, NX210c, for which frequent PK samples were obtained up to 180 min post-dosing [14].

In the multiple ascending dose study (ClinicalTrials.gov Identifier: NCT05827653), healthy elderly subjects (above 54 years of age) were administered 5 mg (*n* = 12) and 10 mg (*n* = 11) NX210c three times weekly for 4 weeks via 10 min infusions. Frequent PK samples were collected on days 1, 12, and 24 [17].

#### 4.4.2. NX210c Mechanism of Action

The effects of NX210c were included in the BBB model guided by in vitro data predictions of expression of TJPs (such as claudin-5), TEER, and BBB permeability compared to no treatment, validated with the observed experimental data [30,51]. In particular, the drug’s MoA targeted BBB integrity through the modulation of critical biomarkers such as endothelial cells’ viability and TJP expression. Within the model, NX210c primarily acted by improving BBB integrity, thereby reducing its leakage and then the probability of immune cell infiltration into the CNS. This upstream effect translated into a clinically relevant outcome, evidenced by a reduction in relapses predominantly driven by alterations in BBB permeability.

### 4.5. Integration of the BBB Model with Multiple Sclerosis Model

To evaluate the potential therapeutic effect of NX210c in RRMS, the BBB model was integrated with MS TreatSim [28,52]. This BBB–RRMS mechanistic coupling and the focus on BBB-targeted therapeutic effects of a new drug already in development allowed generating populations of heterogeneous virtual RRMS patients and predicting the treatment effect on immune cell dynamics and disease progression.

### 4.6. Simulation of Clinical Scenarios

#### 4.6.1. The BBB in Aging Subjects Treated with NX210c

The mechanistic BBB model was first used to illustrate the impact of aging in 40-year-old healthy and RRMS subjects receiving either no treatment or a 4-week NX210c regimen (thrice-weekly), followed by an observation period of 30 years. The simulations explored the effect of NX210c on TJP expression and BBB electrical resistance.

Next, a cohort of subjects were generated and used to simulate two conditions: untreated and treated with repeated administration cycles of 10 mg/kg NX210c every 6 months (thrice-weekly dose for 4 weeks, following the same dosing regimen as in [17]). Subjects were studied for an observation period of 10 years.

#### 4.6.2. Effect of NX210c on Virtual RRMS Patients

We generated a population of 100 virtual patients with heterogeneous disease severity for the prediction of RRMS clinical endpoints such as relapse activity, quantified in the model by oligodendrocyte cell loss. Importantly, heterogeneity in immune repertoire, autoimmune activation propensity, thymic output, and BBB baseline integrity was explicitly encoded via parameter distributions and stochastic processes. Self-antigen injection was inputted to trigger autoimmune responses against oligodendrocytes, resulting in relapses. The threshold for clinical relapse was defined as a 15% reduction in the oligodendrocyte population as in [28]; below this threshold, we consider it as subclinical, i.e., the patient does not manifest clinical symptoms despite new or ongoing inflammatory activity in the CNS. The inclusion/exclusion criteria were set according to the demographic characteristics of the patients enrolled into a typical clinical trial with baseline age, sex, weight, and race. Given the wide variability in predictable MS dynamics stemming from the above-mentioned baseline features, the simulator was run using multiple different immune system repertoires randomly varying the MHC-I major antigens (HLA A, B, and C) and MHC-II locus (DM, DO, DP, DQ, and DR), for each virtual patient. NX210c low (5 mg/kg) and high (10 mg/kg) doses for 4 weeks (thrice-weekly) were compared against no treatment.

Finally, a second virtual patient population was generated based on highly progressing patients. Patients with high disease activity were generated by perturbing model parameters that control risk factors for developing highly active disease such as thymus efficiency, the probability that an autoimmune cell can became active, and parameters associated with HLA-DQ and DR. The effect of 10 mg/kg doses of the NX210c 4-week regimen was tested in monotherapy or in combination with SoC. The MoA of each SoC is detailed in Supplementary Figure S1.

We implemented the following dosing regimens:

- NX210c administration via intravenous infusion: 5 mg/kg or 10 mg/kg thrice-weekly over 4 weeks [14];
- Interferon β-1a administration via subcutaneous self-injection: 22 mcg three times a week [53];
- Teriflunomide administration via oral tablets: 14 mg once a day [28];
- Natalizumab administration via intravenous infusion: 300 mg, once every 4 weeks [28];
- Ocrelizumab administration via intravenous infusion: 600 mg, once every 6 months [54];
- Cladribine administration via oral tablets: 10 mg according to body weight (3.5 mg/kg) over 2 years [55]. The dose is administered by 16–20 tablets taken on the first 5 days of months 1, 2, 13, and 14 of treatment.

## 5. Conclusions

The here-described QSP model simulations explore the longitudinal effects of NX210c on restoring BBB dysfunction (related to natural aging or other pathological factors), with potential application in diseases such as RRMS. Taken together, these results highlight the value of mechanistic QSP modeling in elucidating how BBB-targeted interventions may reshape relapse dynamics at the population level. While the model is not intended for exact precise individual-level patient predictions, it provides a coherent and biologically plausible framework to explore treatment effects, patient heterogeneity, and potential stratification strategies within RRMS drug development programs. This QSP application represents a cost-effective tool for guiding indication selection, supporting the refinement of clinical strategies and optimizing dosing regimens. Ultimately, such approaches can be used for probability of success assessment to inform go/no-go decision-making, and to more quickly help bring effective treatment options to patients.

## Data Availability

Data are not available for the datasets generated and/or analyzed during the current study as they are derived from proprietary software and cannot be made publicly available due to licensing restrictions.

## Supplementary Materials

The following supporting information can be downloaded at: https://www.mdpi.com/article/10.3390/ijms27031349/s1.

## Abbreviations

The following abbreviations are used in this manuscript:

BBB	Blood–brain barrier
BMEC	Brain microvascular endothelial cells
CNS	Central nervous system
MAD	Multiple ascending dose
MoA	Mechanism of action
MS	Multiple sclerosis
MS TreatSim	Multiple Sclerosis Treatment Simulator
QSP	Quantitative systems pharmacology
RRMS	Relapsing–remitting multiple sclerosis
SAD	Single ascending dose
SoC	Standard of care
TEER	Transendothelial electrical resistance
TJP	Tight junction protein
