# Supplementary material for: NX210c Demonstrates Therapeutic Potential to Restore Blood–Brain Barrier in a QSP Model of Relapsing–Remitting Multiple Sclerosis
Source: Int J Mol Sci. 2026 Jan 29;27(3):1349. doi: 10.3390/ijms27031349 (PMC12898804; doi:10.3390/ijms27031349)
Supplement: Supplementary file 1 [file ijms-27-01349-s001.zip › IJMS_Supplementary_Information.pdf]

Supplementary Information

NX210c demonstrates therapeutic potential to restore blood–brain barrier in a QSP model of relapsing–remitting multiple sclerosis

Russo G., Sips F., Catozzi S., Bambury P. et al.

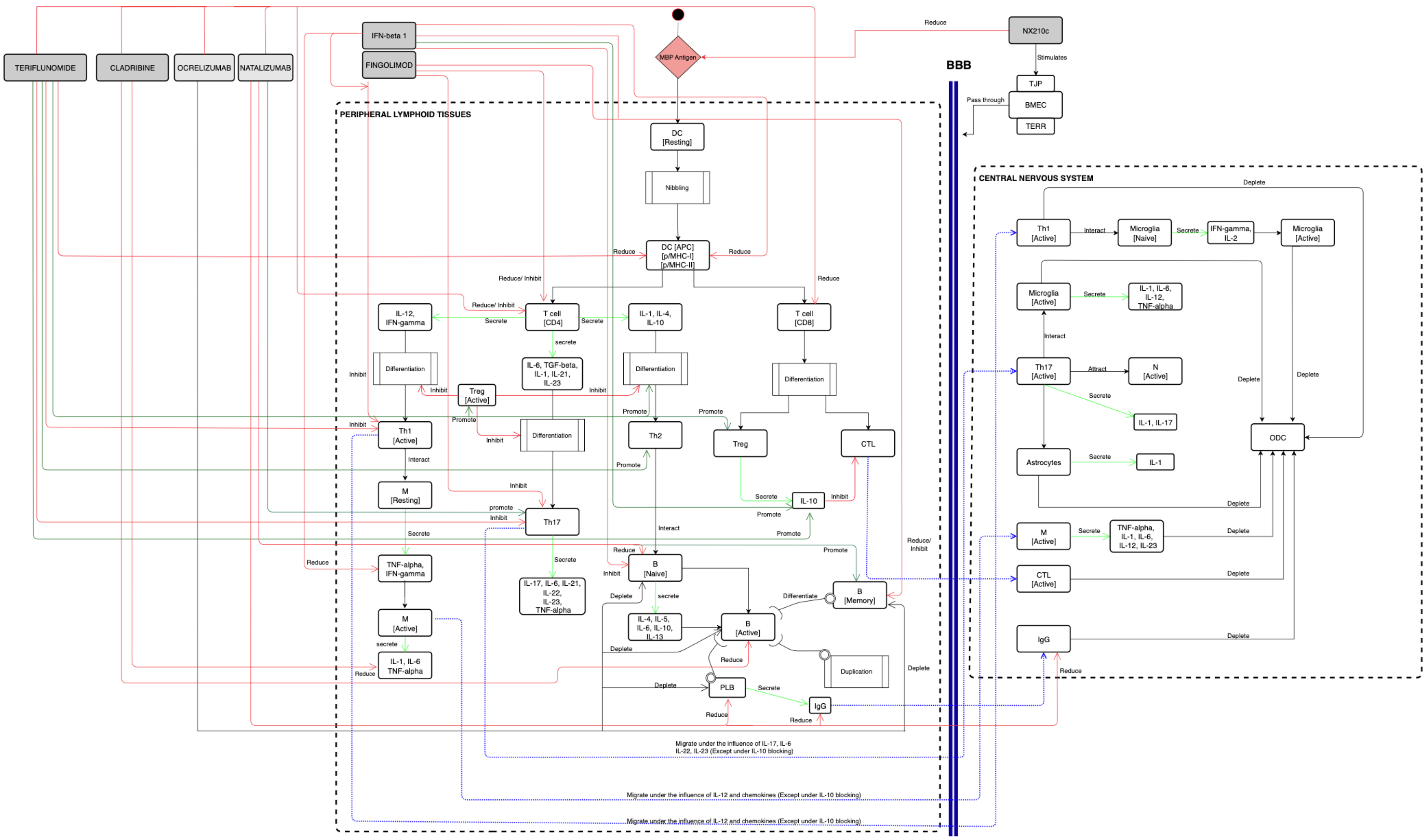

Supplementary Figure S1. The MS–immune system interaction model. Conceptual description of the leading MS entities and interactions and the immune system. The main three compartments, the peripheral lymphoid tissues, the central nervous system and blood-brain barrier, are depicted. The representation describes both cellular and humoral responses. The conceptual model describes also the various treatment layers, including the new NX210c MoA.
